# 60 Running Water While Bathing Is a Risk Factor for Pediatric Scald Burns

**DOI:** 10.1093/jbcr/irac012.063

**Published:** 2022-03-23

**Authors:** William J Moser, Kristen R Bilka, Sebastian Q Vrouwe, Jill Glick, Veena Ramaiah

**Affiliations:** Lurie Children's Hospital, Lombard, Illinois; University of Chicago, Chicago, Illinois; University of Chicago, Chicago, Illinois; university of Chicago, Libertyville, Illinois; University of Chicago Comer Children's Hospital, Chicago, Illinois

## Abstract

**Introduction:**

Scalds are the most common mechanism of burn in children, and a significant proportion of these injuries are associated with bathing. Burns sustained while bathing present a unique opportunity for injury prevention; previous studies have examined lowering water heater temperatures, however reputable infant bathing educational resources do not explicitly recommend avoiding running water and the risks that it could pose. In an effort to inform prevention programs, this study seeks to determine the incidence and circumstances of running water in bathing scald burns at our institution.

**Methods:**

A retrospective review was performed of records from an American Burn Association verified center over a ten year period (1/1/2010 to 12/31/2019). This center treats both children and adults and is affiliated with an academic hospital in a major urban center. The burn database was queried for scald injuries in children less than three years involving bathing. The Child Advocacy and Protective Services team provides inpatient consultation for all children less than three years old with burn injuries allowing us to analyze the specific events surrounding the bathing scald burns in this cohort.

**Results:**

A total of 123 patients met inclusion criteria. Three bathing safety risk factors were specifically noted in the chart review: (1) running water, (2) lack of caregiver presence for duration of bathing, and (3) failure of caregiver to check water temperature before bathing. Of the cases identified, 107 (87%) had clear documentation of running water as part of the history of injury, 66 (54%) cases involved failure of caregiver to check the water temperature before bathing and 53 (43%) cases did not have a caretaker present for the duration of the bath. In cases with only one risk factor, running water was identified in 34 (94%) out of 36 cases, and in cases with one or two risk factors, running water remained the primary risk factor with 38 (90%) out of 42 cases. When looking at the combination of risk factors, only three (2%) cases had no risk factors while 77 (63%) involved two or more risk factors.

**Conclusions:**

The vast majority of bathing burn injuries in this series involved running water. In addition, a significant number of scald burns occurred from running water alone, even without the other identified risk factors. Conversely, only 2% of scald burns associated with bathing featured none of these three risk factors, suggesting that these injuries could be greatly impacted by safe bathing education.

